# Moderating Effect of Coping Strategies on the Association Between the Infodemic-Driven Overuse of Health Care Services and Cyberchondria and Anxiety: Partial Least Squares Structural Equation Modeling Study

**DOI:** 10.2196/53417

**Published:** 2024-04-09

**Authors:** Richard Huan Xu, Caiyun Chen

**Affiliations:** 1 Department of Rehabilitation Sciences Faculty of Health and Social Sciences Hong Kong Polytechnic University Hung Hom China (Hong Kong); 2 Nanjing Academy of Administration Nanjing China

**Keywords:** infodemic, health care, cyberchondria, anxiety, coping, structural equation modeling

## Abstract

**Background:**

The COVID-19 pandemic has led to a substantial increase in health information, which has, in turn, caused a significant rise in cyberchondria and anxiety among individuals who search for web-based medical information. To cope with this information overload and safeguard their mental well-being, individuals may adopt various strategies. However, the effectiveness of these strategies in mitigating the negative effects of information overload and promoting overall well-being remains uncertain.

**Objective:**

This study aimed to investigate the moderating effect of coping strategies on the relationship between the infodemic-driven misuse of health care and depression and cyberchondria. The findings could add a new dimension to our understanding of the psychological impacts of the infodemic, especially in the context of a global health crisis, and the moderating effect of different coping strategies on the relationship between the overuse of health care and cyberchondria and anxiety.

**Methods:**

The data used in this study were obtained from a cross-sectional web-based survey. A professional survey company was contracted to collect the data using its web-based panel. The survey was completed by Chinese individuals aged 18 years or older without cognitive problems. Model parameters of the relationships between infodemic-driven overuse of health care, cyberchondria, and anxiety were analyzed using bootstrapped partial least squares structural equation modeling. Additionally, the moderating effects of coping strategies on the aforementioned relationships were also examined.

**Results:**

A total of 986 respondents completed the web-based survey. The mean scores of the Generalized Anxiety Disorder-7 and Cyberchondria Severity Scale-12 were 8.4 (SD 3.8) and 39.7 (SD 7.5), respectively. The mean score of problem-focused coping was higher than those of emotion- and avoidant-focused coping. There was a significantly positive relationship between a high level of infodemic and increased overuse of health care (bootstrapped mean 0.21, SD 0.03; 95% CI 0.1581-0.271). The overuse of health care resulted in more severe cyberchondria (bootstrapped mean 0.107, SD 0.032) and higher anxiety levels (bootstrapped mean 0.282, SD 0.032) in all the models. Emotion (bootstrapped mean 0.02, SD 0.008 and 0.037, SD 0.015)- and avoidant (bootstrapped mean 0.026, SD 0.009 and 0.049, SD 0.016)-focused coping strategies significantly moderated the relationship between the overuse of health care and cyberchondria and that between the overuse of health care and anxiety, respectively. Regarding the problem-based model, the moderating effect was significant for the relationship between the overuse of health care and anxiety (bootstrapped mean 0.007, SD 0.011; 95% CI 0.005-0.027).

**Conclusions:**

This study provides empirical evidence about the impact of coping strategies on the relationship between infodemic-related overuse of health care services and cyberchondria and anxiety. Future research can build on the findings of this study to further explore these relationships and develop and test interventions aimed at mitigating the negative impact of the infodemic on mental health.

## Introduction

### COVID-19–Related Mental Health Problems

In today’s technologically advancing society, widespread and rapid digitization has led to a substantial increase in the use of social media and the internet. This, in turn, has facilitated the rapid dissemination of all types of information. Although this can be beneficial in filling information gaps quickly, it has its drawbacks. A prominent drawback is the amplification of harmful messages, which can have negative effects on individuals [1,2]. The World Health Organization (WHO) acknowledged the presence of an infodemic during the COVID-19 pandemic and subsequent responses. WHO defines an infodemic as an excessive amount of information, including both accurate and inaccurate content [3]. This abundance of information makes it difficult for individuals to distinguish reliable sources from unreliable sources and to find trustworthy guidance when they need it.

Excessive use of health care services can have adverse effects on individuals and the overall sustainability of health care systems. Although challenges associated with the overuse of health care services were evident before the COVID-19 pandemic [4,5], the urgent need for sustainable health care systems was exacerbated by the pandemic. Because large portions of the population were instructed to self-isolate at home and had limited access to health care professionals during the pandemic, the internet became the primary source of information for numerous individuals seeking answers to health-related questions. However, the abundance of web-based information, including both true and false content, can leave individuals feeling overwhelmed and struggling to make informed choices. This information overload can lead to depression because individuals bombarded with conflicting messages may feel unsure of what to believe [6-10].

Besides depression, cyberchondria has also emerged as a significant public health challenge since the onset of the COVID-19 pandemic. This refers to the repeated and excessive search for health-related information on the internet, leading to a significant increase in distress or anxiety [11]. Although the global emergency caused by the COVID-19 pandemic is over, telehealth remains a growing trend. An increasing number of studies have indicated that telehealth can improve health care access, outcomes, and affordability by offering a bridge to care and an opportunity to reinvent web-based care models [12]. However, increasing internet exposure increases the risk of cyberchondria, especially under conditions of uncertainty and increased risk, due to the large volume of information it contains. Thus, it is crucial to understand how to provide support and guidance to help people adopt appropriate strategies for using web-based resources safely in the context of an infodemic.

### Current Research on the COVID-19–Related Infodemic

The harms of infodemic are well documented. An Italian study suggested developing early warning signals for an infodemic, which can provide important cues for implementing effective communication strategies to mitigate misinformation [13]. Other studies have shown that successful use of coping strategies can help individuals manage stressful events and reduce negative emotions during a pandemic. For example, Yang [14] found a positive correlation between emotion-focused coping and cyberbullying and depression during the COVID-19 pandemic. A large-scale UK study indicated that supportive coping was associated with a faster decrease in depression and anxiety symptoms [15]. Shigeto et al [16] emphasized the importance of training young adults to develop resilience, flexibility, and specific coping skills to offset the psychological effects of significant lifestyle changes resulting from pandemics or other health crises in the future. A recent study used machine learning technology to enhance the accuracy and efficiency of automated fact-checking and infodemic risk management at a strategic level [17]. However, the impact of coping strategies on the relationship among the infodemic, cyberchondria, and anxiety at an individual level during the COVID-19 pandemic is still unknown.

### Importance of Coping Strategies

The ability of individuals to discern and adopt appropriate coping strategies can have a profound impact on their mental health, particularly in relation to conditions such as depression and anxiety. The ability to select and implement coping strategies is not uniform across all individuals, and these differences can significantly influence the trajectory of their mental health outcomes. For some, the ability to effectively choose and implement coping strategies can serve as a protective factor, mitigating the severity of the symptoms of depression or anxiety and promoting overall health and well-being. Conversely, for others, inability or difficulty in selecting and implementing effective coping strategies can exacerbate mental health conditions, leading to increased severity of depression and anxiety. This, in turn, can have detrimental effects on individuals’ overall health and well-being. Therefore, understanding the factors that influence individuals’ ability to select and implement effective coping strategies is of paramount importance in the field of mental health research and intervention [18].

Research has demonstrated the importance of appropriate coping mechanisms in managing mental health problems. Coping strategies, which are essential for dealing with stress or challenging situations, can be categorized into 3 primary types: emotion focused, problem focused, and avoidant focused [19]. Emotion-focused strategies are centered around managing and regulating emotions. They serve as a means to cope with stress or difficult situations. These strategies might involve seeking emotional support from others, using relaxation techniques, or practicing mindfulness. In contrast, problem-focused strategies actively address the problem or stressor. These strategies might encompass problem-solving, devising a plan of action, or seeking information and resources to effectively tackle the situation. Avoidant-focused strategies involve evading or distancing oneself from the stressor or problem. These strategies might include denial, distraction, or engaging in activities to escape or avoid contemplating the issue [18]. The effectiveness of different coping strategies can vary depending on the situation. Individuals often use different or a combination of strategies, tailoring their approach to their circumstances.

### Coping Strategies in the COVID-19–Related Infodemic

From a social perspective, this study underscores the importance of mental health in the context of public health emergencies such as the COVID-19 pandemic. It highlights the need for society to recognize and address the mental health burden that such emergencies can place on individuals, particularly in relation to the phenomenon of cyberchondria, which is the unfounded escalation of concerns about common symptoms based on reviews of web-based literature and resources.

Practically, this study provides valuable insights for policy makers and practitioners. It emphasizes the need for the development of effective coping strategies and programs to manage the negative impact of an overload of misinformation and disinformation on mental health. This is particularly relevant in the digital age, where individuals have access to a plethora of information, not all of which is accurate or reliable. Policy makers and practitioners can use the findings of this study to design interventions that not only provide accurate information but also equip individuals with the skills to distinguish reliable sources from unreliable sources and to cope with the anxiety that misinformation can cause. From a research standpoint, this study fills a gap in the literature by assessing the impact of the infodemic on cyberchondria and the moderating effect of coping strategies in this relationship. It opens up new avenues of research into the complex interplay among public health emergencies, infodemic, cyberchondria, and coping strategies. Future research could build on the findings of this study to further explore these relationships and develop and test interventions aimed at mitigating the negative impact of infodemic on mental health.

### Objective of the Study

Currently, the association between the overuse of health care services and mental health problems in the context of an infodemic remains unclear, as is the moderating effect of different coping strategies on this association. Thus, this study investigated the moderating effect of coping strategies on the relationship between the infodemic-driven misuse of health care and depression and cyberchondria.

### Hypotheses of the Study

The study used a hypothesis-driven format. Specifically, there are five hypotheses: (1) a positive relationship exists between infodemic and the misuse of health care, (2) a positive relationship exists between the misuse of health care and depressive disorders, (3) a positive relationship exists between the misuse of health care and cyberchondria, (4) coping strategies mitigate the negative effect of the misuse of health care on depression, and (5) coping strategies mitigate the negative effect of the misuse of health care on cyberchondria. Hypotheses 2-5 are separately evaluated for the three types of coping strategies: problem focused (H2.1), emotion focused (H2.2), and avoidant focused (H2.3).

## Methods

### Study Design and Sample Size

The data used in this study were obtained from a cross-sectional and web-based survey conducted between April and May 2023 in China.

There is no gold standard for sample estimation in partial least squares structural equation modeling (PLS-SEM). Following Hair et al [20], we set the significance level at 5% and the minimum path coefficients to between 0.05 and 0.1. Based on these criteria, a minimum sample size of 619 was determined.

### Data Source and Collection

A professional surveying company, WenJuanXing, was invited to collect the data through its web-based panel. The panel of WenJuanXing consists of 2.6 million members, with an average of over 1 million questionnaire respondents daily. At the beginning of the project, a survey manager collaborated with the research team to screen and recruit participants using the company’s internal social network platform. All of the eligible panel members received a survey invitation, and a voluntary response sampling method was used. The survey manager checked the data quality using WenJuanXing’s artificial intelligence data quality control system to ensure that respondents met our inclusion criteria and provided valid responses, thus ensuring a high level of data accuracy and integrity. The inclusion criteria were (1) aged older than 18 years, (2) able to understand and read Chinese, and (3) agreed to provide informed consent. All eligible respondents were invited to participate in a web-based survey. The first section of the survey was the informed consent, which the participants were required to read and agree to before proceeding. All the participants who agreed to participate in the survey were asked to complete six questionnaires covering (1) demographics and socioeconomic status, (2) COVID-19 information–related questions, (3) a cyberchondria questionnaire, (4) an eHealth literacy questionnaire, (5) an anxiety questionnaire, and (6) a coping strategy questionnaire. The English translations of the questionnaires are presented in Multimedia Appendix 1. To ensure data quality, we collaborated with the survey company and implemented various indicators. We monitored completion time, excluding responses that took less than 6 minutes. We also tracked ID addresses, ensuring that each ID address could only complete the questionnaire once. To minimize random errors, we used an artificial intelligence formula developed by the survey company to identify and filter any response patterns that appeared to be generated in parallel.

### Ethical Considerations

The study protocol and informed consent process were approved by the institutional review board of the Hong Kong Polytechnic University (HSEARS20230502006). Informed consent was collected from all participants. The survey was conducted anonymously, and no personally identifiable information was collected. No compensation was provided by the research team.

### Instruments

#### Cyberchondria Severity Scale-12

The Cyberchondria Severity Scale-12 (CSS-12), derived from the 33-item CSS, was used to measure the severity of cyberchondria. The CSS-12 exhibited equally good psychometric properties as the original version and has been validated in Chinese populations [21]. The CSS-12 items are scored on a Likert-type scale ranging from 1=“never” to 5=“always,” giving total scores ranging from 12 to 60. A higher score indicates a higher severity of suspected cyberchondria. The psychometric properties of the Chinese version of the CSS-12 were reported by Peng et al [22].

#### Generalized Anxiety Disorder Assessment

The Generalized Anxiety Disorder Assessment-7 (GAD-7) was used to screen for generalized anxiety disorder and related anxiety disorders [23]. This scale consists of 7 items designed to assess the frequency of anxiety symptoms during the 2 weeks preceding the survey. The GAD-7 score is calculated by assigning scores of 0, 1, 2, and 3 to the response categories of “not at all,” “several days,” “more than half the days,” and “nearly every day,” respectively. The scores of the 7 questions are then summed, giving a total ranging from 0 to 21, with higher scores indicating a higher severity of anxiety disorders. Many studies have reported the psychometric properties of the GAD-7 in Chinese populations, such as that conducted by Sun et al [24].

#### Coping Orientation to Problems Experienced Inventory

The Coping Orientation to Problems Experienced Inventory (Brief-COPE) is a 28-item self-report questionnaire used to measure effective and ineffective strategies for coping with a stressful life event [25]. The Brief-COPE assesses how a person deals with stressors in their daily life. The questionnaire measures 3 coping strategy dimensions: problem focused, emotion focused, and avoidant focused [26]. Each item is rated on a 4-point scale. The scores for the 3 overarching coping styles are calculated as average scores. This is done by dividing the sum of the item scores by the number of items. These average scores indicate the extent to which the respondent engages in each coping style. A higher score indicates that the respondent does not have many coping skills. The Chinese version of the Brief-COPE and its psychometric properties in Chinese populations were reported by Wang et al [27].

#### Infodemic- and Misinformation-Driven Overuse of Health Care Services

The COVID-19–related infodemic and misinformation-driven medical misbehavior were assessed using 2 self-developed items. The first item was “Do you believe there is an excessive amount of information regarding the COVID virus and vaccine on a daily basis?” The second item was “Has misinformation or disinformation about COVID-19 led you to engage in the overuse of health care services (eg, frequently visiting the doctor/psychiatrist or buying unnecessary medicine)?” The respondents were required to indicate their response to these 2 questions by selecting 1 of 2 options presented dichotomously: yes or no.

### Statistical Analysis

Descriptive statistics were used to describe the participants’ background characteristics. Continuous variables (eg, age) were calculated as means and SDs. Categorical variables (eg, sex) were calculated as frequencies and proportions. The Pearson correlation coefficient (*r*) was used to examine the association between measures, where *r*≥0.3 and *r*≥0.5 indicated moderate and large effects, respectively [28,29].

In this study, we used PLS-SEM to estimate the research model parameters, as it works efficiently with small samples and complex models. Compared with covariance-based structural equation modeling, PLS-SEM has several advantages, such as the ability to handle non-normal data and small samples [30]. Unlike covariance-based structural equation modeling, which focuses on confirming theories, PLS-SEM is a causal-predictive approach that explains variance in the model’s dependent variables [31]. To improve the model fit, we used the bootstrapping method with 10,000 replications to obtain the estimates of the mean coefficients and 95% CIs [32]. Composite reliability rho_a (>0.7), composite reliability rho_c (>0.7), and average variance extracted (>0.5) were used to examine the model performance.

**Figure 1:**
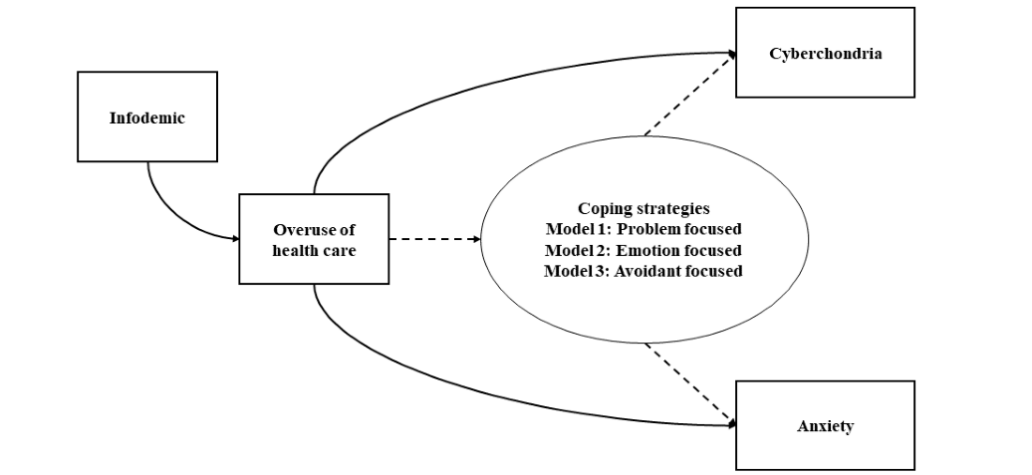
Conceptual framework of this study.

PLS-SEM encompasses measurement models that define the relationship between constructs (instruments) and indicator variables and a structural model. The structural model used in this study is presented in Figure 1. We hypothesized that the infodemic significantly affects misinformation-driven medical misbehavior, resulting in cyberchondria and high anxiety levels. Furthermore, we speculated that coping strategies significantly modify this relationship. To test these hypotheses, we used 3 models that used the full sample to separately investigate the moderating effect of the 3 types of coping strategies (problem focused, emotion focused, and avoidant focused). We analyzed the data and estimated the PLS-SEM parameters using the “SEMinR” package in R (R Foundation for Statistical Computing). A *P* value of ≤.05 was considered statistically significant.

## Results

### Background Characteristics of Participants

A total of 986 respondents completed the web-based survey and provided valid responses, resulting in a response rate of 84%. Among the participants, 51.7% (n=510) were female, approximately 95% (n=933) had completed tertiary education or above, and 71.2% (n=702) resided in urban areas. The participants’ background characteristics are listed in Table 1.

**Table 1:** Respondents’ background characteristics (n=986).

			Respondents, n (%)
**Sex**			
	Male	476 (48.3)	
	Female	510 (51.7)	
**Educational level**			
	Secondary or below	53 (5.4)	
	Tertiary or above	933 (94.6)	
**Household registry**			
	Urban	702 (71.2)	
	Rural	284 (28.8)	
**Employment**			
	Active	894 (90.7)	
	Nonactive	92 (9.3)	
**Marital status**			
	Single	232 (23.5)	
	Married	750 (76.1)	
	Divorce or widowed	4 (0.4)	
**Family annual income (CNY)^a^**			
	≤50,000	57 (5.8)	
	50,001-100,000	191 (19.4)	
	100,001-200,000	341 (34.6)	
	200,001-300,000	249 (25.3)	
	300,001-400,000	89 (9)	
	> 400,000	59 (6)	
**Diagnosed with chronic disease**			
	Yes	321 (32.6)	
	No	665 (67.4)	

^a^A currency exchange rate of 7.23 CNY=US $1 applies.

### Mean Scores and Frequency of Responses

The mean score of the GAD-7 was 8.4 (SD 3.8), while the mean score of the CSS-12 was 39.7 (SD 7.5). Problem-focused coping had a higher mean score than emotion- and avoidant-focused coping. Respondents with active employment reported statistically significantly higher mean scores on the GAD and avoidant-focused coping subscale compared to those with nonactive employment. A higher proportion of respondents with chronic diseases experienced an infodemic and exhibited the overuse of health care services relative to those without chronic diseases (Table 2). The correlations between all of the measures are presented in Multimedia Appendix 2.

**Table 2:** Mean score of GAD-7^a^, CSS-12^b^, Brief-COPE^c^, frequency of infodemic, and overuse of health care in different groups of background characteristics (n=986).

		Infodemic, yes, n (%)	Overuse of health care, mean (SD)	GAD-7, mean (SD)	CSS-12, mean (SD)	Brief-COPE, mean (SD)		
						Problem	Emotion	Avoidant
Overall		581 (58.9)	596 (60.4)	8.4 (3.8)	39.7 (7.5)	3.3 (0.4)	2.6 (0.3)	2.1 (0.5)
**Sex**								
	Male	283 (59.5)	282 (59.2)	8.2 (3.7)	39.8 (7.6)	3.3 (0.5)	2.6 (0.4)	2.1 (0.5)
	Female	298 (58.4)	314 (61.6)	8.6 (3.8)	39.6 (7.4)	3.3 (0.4)	2.6 (0.3)	2.1 (0.5)
**Educational level**								
	Secondary or below	32 (59.6)	29 (55.8)	9 (4.5)	37.3 (7.8)^d^	3.2 (0.5)	2.6 (0.4)	2.2 (0.5)
	Tertiary or above	549 (58.8)	567 (60.8)	8.3 (3.7)	39.9 (7.5)	3.3 (0.4)	2.6 (0.3)	2.1 (0.4)
**Family registry**								
	Urban	428 (61)^d^	430 (61.3)	8.1 (3.7)^e^	40 (7.6)^f^	3.3 (0.5)	2.6 (0.3)	2.1 (0.5)
	Rural	153 (53.9)	166 (58.5)	9 (4)	39 (7.1)	3.2 (0.4)	2.7 (0.3)	2.2 (0.5)
**Employment**								
	Active	525 (58.7)	557 (62.3)^g^	9.8 (4.2)^g^	38.1 (7.3)^h^	3.2 (0.4)	2.6 (0.3)	2.2 (0.5)^i^
	Nonactive	56 (60.9)	39 (42.4)	8.2 (3.7)	40 (7.5)	3.3 (0.5)	2.6 (0.3)	2.1 (0.4)
**Chronic disease**								
	Yes	208 (64.8)^j^	239 (74.5)^g^	9.1 (4)^g^	41.2 (6.6)^g^	3.2 (0.4)	2.7 (0.3)^k^	2.2 (0.5)^g^
	No	373 (56.1)	357 (53.7)	8 (3.7)	39 (7.8)	3.3 (0.5)	2.6 (0.3)	2.0 (0.4)

^a^GAD-7: Generalized Anxiety Disorder Assessment-7.

^b^CSS-12: Cyberchondria Severity Scale-12.

^c^COPE: Coping Orientation to Problems Experienced Inventory.

^d^*P*=.04.

^e^*P*=.002.

^f^*P*=.05.

^g^*P*<.001.

^h^*P*=.03.

^i^*P*=.02.

^j^*P*=.009.

^k^*P*=.01.

### Measurement Models

Tables 3-5 present the performance of the measurement models for the 3 coping strategies. The values of rho_C and rho_A were above 0.7, indicating acceptable construct reliability. All 3 constructs had Cronbach α values exceeding the cutoff of 0.7, indicating adequate reliability. Table 2 presents the models’ convergent validity. All the bootstrapped item loadings exceeded 0.3 and were significant at <.05 for the problem- and avoidant-focused models. However, for cyberchondria and the Brief-COPE, none of the average variance extracted values were above 0.5, indicating unsatisfactory model convergent validity.

**Table 3:** Performance of the measurement model: problem-focused model.

			Bootstrapped loadings (95% CI)		Cronbach α		rho_C		AVE^a^		rho_A
**GAD-7^b^**					0.842		0.878		0.505		0.865
	GAD1	0.737 (0.692-0.774)									
	GAD2	0.772 (0.738-0.804)									
	GAD3	0.712 (0.661-0.755)									
	GAD4	0.637 (0.58-0.688)									
	GAD5	0.714 (0.665-0.756)									
	GAD6	0.641 (0.584-0.689)									
	GAD7	0.759 (0.721-0.794)									
**Cyberchondria**					0.833		0.865		0.350		0.834
	CYB1	0.581 (0.514-0.638)									
	CYB2	0.559 (0.427-0.65)									
	CYB3	0.599 (0.538-0.653)									
	CYB4	0.632 (0.54-0.693)									
	CYB5	0.539 (0.362-0.678)									
	CYB6	0.661 (0.609-0.703)									
	CYB7	0.562 (0.432-0.651)									
	CYB8	0.649 (0.56-0.709)									
	CYB9	0.635 (0.537-0.704)									
	CYB10	0.481 (0.344-0.581)									
	CYB11	0.58 (0.458-0.66)									
	CYB12	0.534 (0.374-0.655)									
**Brief-COPE^c^**					0.770		0.831		0.387		0.791
	BF2	0.723 (0.668-0.773)									
	BF7	0.729 (0.683-0.771)									
	BF10	0.453 (0.34-0.546)									
	BF12	0.67 (0.611-0.723)									
	BF14	0.669 (0.61-0.72)									
	BF17	0.567 (0.499-0.629)									
	BF23	0.463 (0.345-0.554)									
	BF25	0.614 (0.55-0.671)									
	BF19	0.723 (0.668-0.773)									
**Infodemic**											
	IF1	1.0 (1.0-1.0)		1.0		1.0		1.0		1.0	
**Overuse of HC^d^**											
	OHC1	1.0 (1.0-1.0)		1.0		1.0		1.0		1.0	

^a^AVE: average variance extracted.

^b^GAD-7: Generalized Anxiety Disorder-7.

^c^COPE: Coping Orientation to Problems Experienced Inventory.

^d^HC: health care.

**Table 4:** Performance of the measurement model: emotion-focused model.

			Bootstrapped loadings (95% CI)		Cronbach α			rho_C			AVE^a^				rho_A		
**GAD-7^b^**						0.842			0.880			0.512				0.848	
	GAD1	0.755 (0.72 to 0.787)															
	GAD2	0.752 (0.716 to 0.784)															
	GAD3	0.726 (0.683 to 0.763)															
	GAD4	0.648 (0.596 to 0.692)															
	GAD5	0.719 (0.678 to 0.757)															
	GAD6	0.677 (0.632 to 0.718)															
	GAD7	0.723 (0.682 to 0.758)															
**Cyberchondria**						0.833			0.861			0.349				0.845	
	CYB1	0.565 (0.5 to 0.625)															
	CYB2	0.64 (0.593 to 0.682)															
	CYB3	0.555 (0.487 to 0.616)															
	CYB4	0.695 (0.654 to 0.731)															
	CYB5	0.36 (0.252 to 0.454)															
	CYB6	0.65 (0.601 to 0.693)															
	CYB7	0.66 (0.615 to 0.7)															
	CYB8	0.686 (0.643 to 0.725)															
	CYB9	0.704 (0.664 to 0.739)															
	CYB10	0.574 (0.515 to 0.624)															
	CYB11	0.465 (0.375 to 0.542)															
	CYB12	0.385 (0.282 to 0.472)															
**Brief-COPE^c^**						0.764			0.718			0.381				0.732	
	BF5	0.182 (0.028 to 0.325)															
	BF9	0.2 (0.058 to 0.329)															
	BF13	0.594 (0.519 to 0.669)															
	BF15	0.13 (–0.026 to 0.279)															
	BF18	0.316 (0.205 to 0.415)															
	BF20	–0.219 (–0.332 to–0.096)															
	BF21	0.609 (0.541 to 0.666)															
	BF22	0.552 (0.449 to 0.643)															
	BF24	–0.28 (–0.394 to –0.142)															
	BF26	0.666 (0.605 to 0.719)															
	BF27	0.586 (0.508 to 0.651)															
	BF28	–0.111 (–0.248 to 0.026)															
**Infodemic**																	
	IF1	1.0 (1.0 to 1.0)		1.0			1.0						1.0				1.0
**Overuse of HC^d^**																	
	OHC1	1.0 (1.0 to 1.0)		1.0			1.0						1.0				1.0

^a^AVE: average variance extracted.

^b^GAD-7: Generalized Anxiety Disorder-7.

^c^COPE: Coping Orientation to Problems Experienced Inventory.

^d^HC: health care.

**Table 5:** Performance of the measurement model: avoidant-focused model.

		Bootstrapped loadings (95% CI)	Cronbach α	rho_C	AVE^a^	rho_A
**GAD-7^b^**			0.842	0.879	0.511	0.854
	GAD1	0.750 (0.712-0.785)				
	GAD2	0.763 (0.732-0.794)				
	GAD3	0.714 (0.671-0.752)				
	GAD4	0.643 (0.591-0.689)				
	GAD5	0.716 (0.673-0.754)				
	GAD6	0.666 (0.62-0.708)				
	GAD7	0.740 (0.703-0.772)				
**Cyberchondria**			0.833	0.861	0.348	0.842
	CYB1	0.566 (0.499-0.624)				
	CYB2	0.652 (0.607-0.692)				
	CYB3	0.552 (0.489-0.609)				
	CYB4	0.684 (0.642-0.724)				
	CYB5	0.351 (0.247-0.446)				
	CYB6	0.644 (0.593-0.688)				
	CYB7	0.661 (0.618-0.702)				
	CYB8	0.686 (0.645-0.724)				
	CYB9	0.692 (0.651-0.726)				
	CYB10	0.597 (0.544-0.643)				
	CYB11	0.460 (0.369-0.538)				
	CYB12	0.384 (0.289-0.471)				
**Brief-COPE^c^**			0.710	0.796	0.346	0.749
	B1	0.372 (0.179-0.364)				
	BF3	0.558 (0.491-0.618)				
	BF4	0.653 (0.595-0.699)				
	BF6	0.647 (0.593-0.695)				
	BF8	0.672 (0.621-0.715)				
	BF11	0.690 (0.636-0.734)				
	BF16	0.712 (0.669-0.751)				
	BF19	0.313 (0.216-0.395)				
**Infodemic**						
	IF1	1.0 (1.0-1.0)	1.0	1.0	1.0	1.0
**Overuse of HC^d^**						
	OHC1	1.0 (1.0-1.0)	1.0	1.0	1.0	1.0

^a^AVE: average variance extracted.

^b^GAD-7: Generalized Anxiety Disorder.

^c^COPE: Coping Orientation to Problems Experienced Inventory.

^d^HC: health care.

### Structural Models

The structural model analysis involved estimating path coefficients for the conceptual model. We performed PLS-SEM on the research model 3 times to estimate path coefficients for the models with different coping strategies. We found that H1 was supported. A significant and positive relationship was observed between a high level of infodemic exposure and increased overuse of health care services (coefficient=0.212, 95% CI 0.151-0.271). In addition, the overuse of health care services was correlated with more severe cyberchondria and higher anxiety levels in all the 3 models, supporting H2 and H3. The effect of the overuse of health care services on cyberchondria was larger than its effect on anxiety. All these relationships were statistically significant (Tables 3-5).

### Moderating Effects

**Figure 2:**
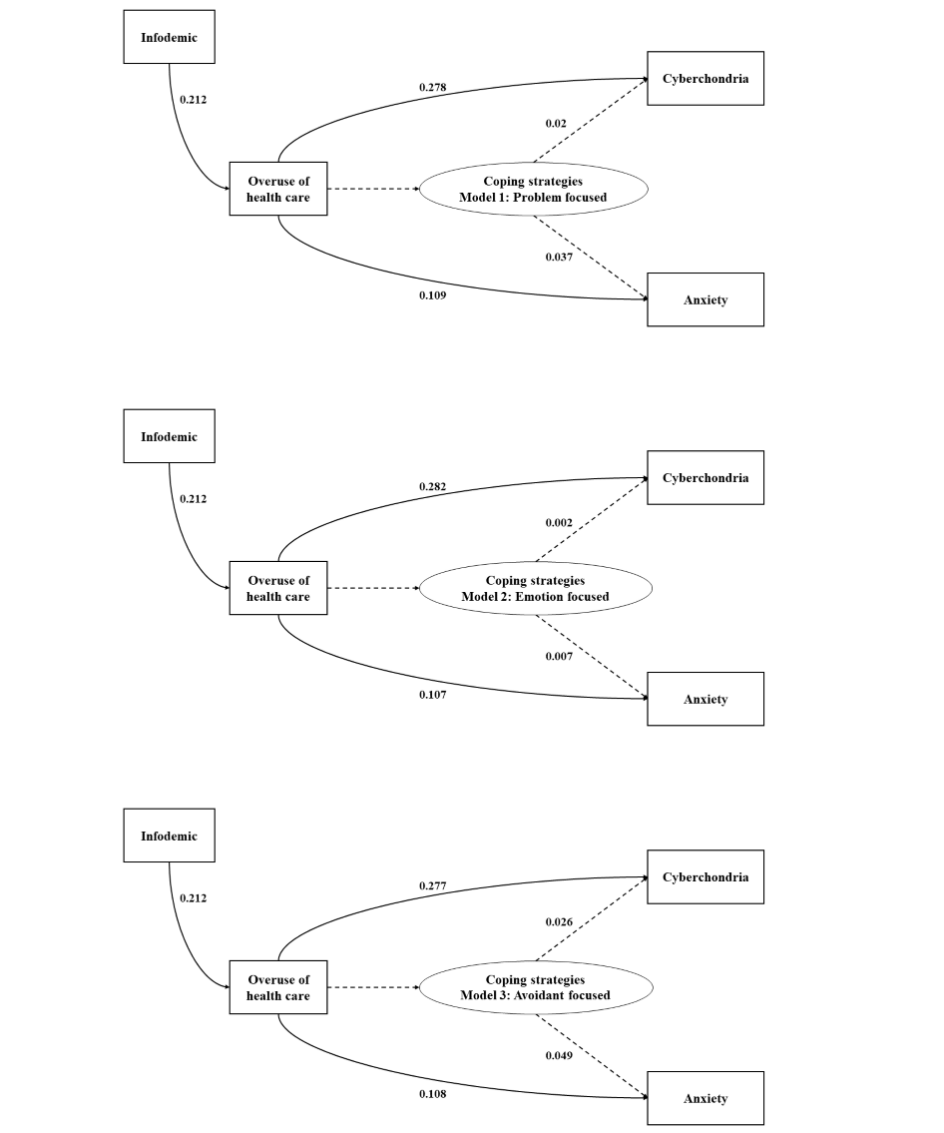
Conceptual framework with partial least squares structural equation modeling estimates.

In our moderation analyses (Figure 2 and Tables 6 and 7), we found that emotion- and avoidant-focused coping strategies significantly moderated the relationship between the overuse of health care services and cyberchondria and that between the overuse of health care services and anxiety, respectively, supporting H5 and H6. For the problem-based model (H4), the moderating effect was not significant for the relationship between the overuse of health care services and cyberchondria (coefficient=0.002, 95% CI −0.011 to 0.006), indicating that H4.1 was not supported. Compared with the direct effects on the relationship between the overuse of health care services and cyberchondria or anxiety, a strong ability to cope with difficulties can effectively mitigate the negative effects of the infodemic-driven overuse of health care services on cyberchondria and anxiety.

**Table 6:** Direct effects for the models.

Relationship			Bootstrapped mean (SD)		95% CI		Hypothesis		Responses
Infodemic→Overuse of HC^a^			0.212 (0.032)		0.151-0.271		H1		Yes
**Problem-focused model**									
	Overuse of HC→Cyberchondria	0.282 (0.032)		0.215-0.338		H2.1		Yes	
	Overuse of HC→GAD^b^	0.107 (0.032)		0.044-0.171		H3.1		Yes	
**Emotion-focused model**									
	Overuse of HC→Cyberchondria	0.278 (0.031)		0.210-0.337		H2.2		Yes	
	Overuse of HC→GAD	0.109 (0.032)		0.046-0.172		H3.2		Yes	
**Avoidant-focused model**									
	Overuse of HC→Cyberchondria	0.277 (0.031)		0.208-0.336		H2.3		Yes	
	Overuse of HC→GAD	0.108 (0.032)		0.046-0.171		H3.3		Yes	

^a^HC: health care.

^b^GAD: Generalized Anxiety Disorder Assessment.

**Table 7:** Moderating effects of the coping strategies.

Relationship			Bootstrapped mean (SD)		95% CI		Hypothesis		Responses
**Problem-focused model**									
	Overuse of HC^a^→CS^b^→Cyberchondria	0.002 (0.004)		–0.011 to 0.006		H4.1		No	
	Overuse of HC→CS→GAD^c^	0.007 (0.011)		0.005 to 0.027		H5.1		Yes	
**Emotion-focused model**									
	Overuse of HC→CS→Cyberchondria	0.020 (0.008)		0.004 to 0.039		H4.2		Yes	
	Overuse of HC→CS→GAD	0.037 (0.015)		0.008 to 0.066		H5.2		Yes	
**Avoidant-focused model**									
	Overuse of HC→CS→Cyberchondria	0.026 (0.009)		0.010 to 0.044		H4.3		Yes	
	Overuse of HC→CS→GAD	0.049 (0.016)		0.019 to 0.081		H5.3		Yes	

^a^HC: health care.

^b^CS: coping strategy.

^c^GAD: Generalized Anxiety Disorder Assessment.

## Discussion

### Principal Findings

We performed a series of PLS-SEM analyses to examine the relationships between the infodemic-driven overuse of health care services and cyberchondria and anxiety and determine the moderating effects of 3 types of coping strategies on these relationships. We observed that the individuals who were exposed to an overload of COVID-19–related information were more likely to seek and use extra and unnecessary health care services during the pandemic. Such behavior may lead to a considerable wastage of health resources that are particularly limited during a public health crisis. Although some studies have indicated that during the COVID-19 pandemic individuals with increasing mental health symptoms rarely used mental health services [33-35], we found that the overuse of health care services may contribute to higher levels of depression and cyberchondria during a pandemic. This finding has never been reported before. However, we did not differentiate between the types of health care services, either physical or mental, that the individuals overused during the pandemic. This limitation may affect the implications of our findings for policy making purposes.

### Comparisons With Previous Studies

We observed that enhanced coping strategies can mitigate the adverse effects of overusing health care on depression and cyberchondria. Studies have confirmed the association between pandemics and depression, have identified several sources of depression [6,7,10,36,37], and have determined the relationship between depression and cyberchondria [38]. However, few studies have investigated the relationship between depression or cyberchondria and the infodemic-driven overuse of health care services. Our findings demonstrate that the adverse effects of the pandemic are diverse and require the investigation of individuals’ health from multiple perspectives (ie, infodemic in health communication, the use of health care in health service research, and depression in psychiatry). These effects might not be immediately apparent, but they are all linked to each other and collectively cause harm. Thus, policy makers should develop a comprehensive and cost-effective strategy to address the potential adverse effects of pandemics on people’s health and well-being and better prepare for the next public health crisis.

This study offers new insights into the role of coping strategies in mediating the relationship between health care overuse and depression or cyberchondria during the COVID-19 pandemic. Overall, individuals with strong coping abilities were more likely to report lower levels of depression or cyberchondria than those with weak coping abilities. However, the moderating effects of different coping strategies varied slightly. We discovered that problem-focused coping strategies resulted in lower levels of depression and cyberchondria than avoidant-focused coping strategies. Additionally, emotion-focused coping strategies led to lower levels of depression than the other 2 types of coping strategies. These findings partially align with previous studies. For instance, Li [39] demonstrated that using both problem-focused and emotion-focused coping strategies was beneficial for psychological well-being. However, previous studies have reported mixed findings. For example, AlHadi et al [40] indicated that emotion-focused coping strategies were associated with increased depression, anxiety, and sleep disorders in the Saudi Arabian population. Few studies have examined the effect of avoidant-focused coping strategies. In this study, we found that respondents who reported living with chronic diseases exhibited a higher ability to use avoidant-focused coping. This finding is partially consistent with a previous study that found a positive relationship between avoidance-focused coping strategies and mental health in women with heart disease [41]. Individuals with medical conditions are more likely to adopt avoidant coping strategies. Firouzbakht et al [42] explained that avoidance is an effective strategy for handling short-term stress and is more likely to be adopted by certain patient groups.

We found that individuals who favor emotion-focused coping strategies to overcome difficulties are able to effectively mitigate the adverse effects of excessive health care use on depression and cyberchondria relative to those who opt for the other 2 coping strategies. This finding is not entirely surprising or unexpected. It is, in fact, quite reasonable when one considers that scholars and researchers in the field have previously indicated that people have a tendency to adopt emotion-focused strategies, especially when they find themselves in situations that are uncontrollable or unpredictable, such as the ongoing global pandemic [43]. Some studies have found that age can have a significant impact on an individual’s coping strategy preferences. For instance, younger adults were more likely to use emotion-focused coping strategies during the acute phase of the SARS outbreak, whereas older adults used this particular strategy several months after the outbreak had initially occurred [44]. This suggests that the coping strategies adopted by individuals can vary greatly depending on their age and the stage of the crisis they are experiencing. However, in the context of this study, we did not observe any significant differences in the coping strategy preferences of the different age groups. This could be due to a variety of factors, but a possible explanation is that our model incorporated the COVID-19 infodemic. In this context, it is understandable that providing emotional support might be more important than providing real solutions. This is particularly true in the current digital age, where the internet offers unlimited information sources for people to explore, which can often lead to information overload and increased anxiety. Therefore, emotion-focused coping strategies could be more beneficial in helping individuals navigate the sea of information and manage their emotional responses effectively.

In this study, we used self-developed items to measure the infodemic and overuse of health care services. While this approach allowed us to collect data that were directly related to the research questions, it may have introduced some potential issues. First, self-developed items may have less validity and reliability than standardized questionnaires. This could affect the accuracy of measurements and the validity of findings. Second, using self-developed items may limit comparability with other studies that use standardized questionnaires. Standardized questionnaires allow for easy comparison across studies and populations. The lack of a common metric may make it challenging to compare the findings of this study to other studies or to aggregate them in future meta-analyses. Finally, self-developed items may be more susceptible to response bias. They may not have considered factors like social desirability bias or acquiescence bias as standardized questionnaires do. This could have skewed the responses and affected the accuracy of the findings. Despite these limitations, the study’s findings provide valuable insights and pave the way for future research in this area.

### Main Contributions of This Study

The importance of preparedness, prevention, and emergency response to infodemiology is highly encouraged by the WHO [45]. This study makes a significant contribution by exploring and empirically evaluating the relationship between the infodemic, the overuse of health care services, cyberchondria, and anxiety in the context of the COVID-19 pandemic. It provides empirical evidence supporting the assertion that a high level of infodemic can lead to the increased overuse of health care services, resulting in more severe cyberchondria and heightened anxiety levels. This finding adds a new dimension to our understanding of the psychological impacts of the infodemic, especially in the context of a global public health crisis. Additionally, this study highlights that adopting appropriate coping strategies can potentially reduce the severity of cyberchondria and anxiety, even among people facing high levels of the infodemic and the overuse of health care services.

### Future Research

The study’s findings emphasize the importance of coping strategies in reducing the negative effects of the infodemic and the excessive use of health care. Future research could focus on developing and testing interventions to improve coping skills, such as cognitive-behavioral, mindfulness-based, or psychoeducational approaches. Additionally, other factors like social support, personality traits, or health literacy may moderate the relationship between infodemic, health care overuse, cyberchondria, and anxiety. Future research could further explore these variables. This study’s findings may not apply to all populations, so future research could investigate these relationships in different groups, including those with pre-existing mental health conditions, health care professionals, or diverse cultural contexts. By pursuing these future directions, researchers could build on this study’s findings, thereby enhancing our understanding of the psychological impact of infodemic and developing effective interventions.

### Limitations

This study has several limitations that need to be addressed. A primary limitation is that the data were cross-sectional and self-reporting, which can introduce several biases. Social desirability bias may occur when respondents provide answers they believe are socially acceptable rather than truthful. Recall bias may also be present, as the respondents were asked to recall experiences from months or even a year ago. The data are also prone to response bias, as respondents may agree or disagree with statements regardless of their content. These biases may have affected the accuracy of the findings. In the future, we will try to collect data at multiple time points to reduce the biases and identify changes over time. Second, the data used in this analysis were obtained from a web-based survey, which excluded individuals who are not familiar with web-based surveys or do not have access to the internet. This could have resulted in selection bias. Additionally, due to the nature of the web-based survey, the demographic information of our sample was highly skewed. The majority of the respondents were young and highly educated and were frequent internet users who may have experienced more infodemic effects than older and less educated individuals. This may have affected the reliability of our findings. A quota sampling method could be used in future studies to improve the representativeness of the sample. Third, the study was conducted in China; thus, it is important to consider the unique context of China when interpreting the results. It is necessary to conduct further research in different cultural and regional contexts to determine the generalizability of the results. Finally, the evaluation of health care service overuse and strength of the infodemic relied on 2 self-developed items, which may have affected the measurement properties and limited the reliability of our findings. The development of standardized questionnaires to measure the infodemic and the overuse of health care services during a pandemic would be a valuable contribution to future research in this field.

### Conclusions

This study is the first to demonstrate a significant correlation between the infodemic-driven overuse of health care services and high levels of depression and cyberchondria in the Chinese population during the COVID-19 pandemic. We find that 3 types of coping strategies can effectively mitigate the adverse effects of infodemic-driven health care overuse on depression and cyberchondria. Among them, emotion-focused coping strategies have stronger moderating effects than the other 2 types of coping strategies. These findings provide empirical evidence that can guide policy makers in developing strategies to reduce cyberchondria, provide accurate information about public health crises, and promote adaptive coping strategies to effectively manage future public health crises.

## Appendix

Multimedia Appendix 1 English-translated questionnaire.

Multimedia Appendix 2 Correlations between measures.

## Abbreviations

Brief-COPE: Coping Orientation to Problems Experienced Inventory
CSS-12: Cyberchondria Severity Scale-12
GAD-7: Generalized Anxiety Disorder Assessment-7
PLS-SEM: partial least squares structural equation modeling
WHO: World Health Organization

## Data Availability

The data sets generated and analyzed during this study are available from the corresponding author on reasonable request.
