# Supplementary material for: Moderating Effect of Coping Strategies on the Association Between the Infodemic-Driven Overuse of Health Care Services and Cyberchondria and Anxiety: Partial Least Squares Structural Equation Modeling Study
Source: J Med Internet Res. 2024 Apr 9;26:e53417. doi: 10.2196/53417 (PMC11040441; doi:10.2196/53417)
Supplement: Multimedia Appendix 1 [file jmir_v26i1e53417_app1.docx]

**Research on Internet health literacy and mental health**

We are conducting a research on "Internet Health Literacy and Mental Health", and we sincerely invite you to participate in this survey, and your opinions will provide an important basis for our research.

**Informed Consent Statement:**

This survey has been approved by the University Ethics Committee, and we are committed to keeping the data collected strictly confidential and using it for scientific research purposes only. **By submitting a survey, you are aware of all the contents of the survey and the purpose of the survey, and that you are willing to participate in the study.** During this process, you have the right to terminate your participation in this survey at any time.

Part 1 Personal Information

| 1 Gender:  □1 male,  □2 female |
| --- |
| 2 Year of birth (e.g., 1970): ____________________ |
| 3Education level  □1 Elementary school and below  □2 Middle school (including junior high school and high school)  □ 3. University or above |
| 4. Household registration  □1. Urban household registration  □2. Rural household registration |
| 5. Do you live in your domicile  □1 Yes  □2 No |
| 6. Working conditions:  □1, full-time work,  □2, part-time work,  □3, retirement,  □4, unemployed,  □5, housewife |
| 7. Types of medical insurance:  □1 Urban employee insurance  □2. Urban resident insurance  □3 NCMS  □4. No insurance |
| 8 Marital Status  □ Single  □ Married  □ Divorced/Widowed |
| 9. Disposable annual household income:  □ 1 ≤ 50,000 □ 2 50,001~100,000  □ 3,100,001~200,000 □ 4,200,001~300,000  □ 5,300,001~400,000 □6 >,400,000 |
| 10 Have you been diagnosed with a chronic illness  □ Yes  □ No |

Part 2 Information on the Coronavirus

| 1. Do you feel that there is too much information about the coronavirus in your surroundings?  □Yes  □No |
| --- |
| 2. Have you received/read false information about the coronavirus?  □Yes  □No |
| 3 Have you received/read false information about the COVID vaccine?  □Yes  □No |
| 4. Do you think this false information has caused you some trouble in your life?  □Yes  □No |
| 5. Does this COVID misinformation cause you to over-the-top medical practices (e.g. hoarding medicines)  □Yes  □No |
| 6 Do you think the government has effectively regulated disinformation?  □Yes  □No  □Not sure |
| 7 Do you think it is important to effectively regulate coronavirus or vaccine misinformation?  □Important  □Not important |
| 8. Have you ever been infected with the coronavirus?  □ Yes  □ No |

Part 3 Please read the following statements and indicate how they typically apply to you by circling the appropriate number. Please note that this questionnaire relates to perceived medical conditions (i.e., conditions you think you might have) rather than conditions that have been diagnosed by a medical profession.

|  | never | seldom | sometimes | often | always |
| --- | --- | --- | --- | --- | --- |
| 1. If I notice an unexplained bodily sensation I will search for it on the internet |  |  |  |  |  |
| 1. Researching symptoms or perceived medical conditions online distracts me from reading news/ sports/ entertainment articles online |  |  |  |  |  |
| 1. I read different web pages about the same perceived condition |  |  |  |  |  |
| 1. I start to panic when I read online that a symptom I have is found in a rare/serious condition |  |  |  |  |  |
| 1. Researching symptoms or perceived medical conditions online leads me to consult with my GP |  |  |  |  |  |
| 1. I enter the same symptoms into a web search on more than one occasion |  |  |  |  |  |
| 1. Researching symptoms or perceived medical conditions online interrupts my work (e.g., writing emails, working on word documents or spreadsheets) |  |  |  |  |  |
| 1. I think I am fine until I read about a serious condition online |  |  |  |  |  |
| 1. I feel more anxious or distressed after researching symptoms or perceived medical conditions online |  |  |  |  |  |
| 1. Researching symptoms or perceived medical conditions online interrupts my offline social activities (e.g., reduces time spent with friends/family) |  |  |  |  |  |
| 1. I suggest to my GP/medical professional that I may need a diagnostic procedure that I read about online (e.g., a biopsy/a specific blood test) |  |  |  |  |  |
| 1. Researching symptoms or perceived medical conditions online leads me to consult with other medical specialists (e.g., consultants) |  |  |  |  |  |

Part 4 We would like to learn more about your experience using the Internet to access health information. For the following statements, choose the one that best describes your situation.

|  |  | **Very**  **disagree** | **Disagree** | **Not sure** | **Agree** | **Very**  **agree** |
| --- | --- | --- | --- | --- | --- | --- |
| 1 | I know what kind of health information can be found on the Internet |  |  |  |  |  |
| 2 | I know where to find useful health information online |  |  |  |  |  |
| 3 | I know how to find useful information on the Internet |  |  |  |  |  |
| 4 | I know how to use the internet to find answers to the health questions I want to know |  |  |  |  |  |
| 5 | I know how to use the health information I find on the internet to solve problems |  |  |  |  |  |
| 6 | I have the necessary skills that will allow me to assess the usefulness of the information I find on the internet |  |  |  |  |  |
| 7 | I can distinguish between the high and low quality of health information on the Internet |  |  |  |  |  |
| 8 | I can confidently use the information I find on the internet to make health decisions |  |  |  |  |  |

Part 5

|  | How often in the past two weeks have you been plagued by the following questions? | Not at all | Few days | More than half the days | Almost every day |
| --- | --- | --- | --- | --- | --- |
| 1 | Feeling nervous, anxious, or on edge | □0 | □1 | □2 | □3 |
| 2 | Not being able to stop or control worrying | □0 | □1 | □2 | □3 |
| 3 | Worrying too much about different things | □0 | □1 | □2 | □3 |
| 4 | Trouble relaxing | □0 | □1 | □2 | □3 |
| 5 | Being so restless that it is hard to sit still | □0 | □1 | □2 | □3 |
| 6 | Becoming easily annoyed or irritable | □0 | □1 | □2 | □3 |
| 7 | Feeling afraid, as if something awful might happen | □0 | □1 | □2 | □3 |

Part 6 The following questions ask how you have sought to cope with a hardship in your life. Read the statements and indicate how much you have been using each coping style.

| How you react when faced with a difficult situation or a stressful event | I haven't been doing this at all | A little bit | A medium amount | I’ve been doing this a lot |
| --- | --- | --- | --- | --- |
| I've been turning to work or other activities to take my mind off things. | 1 | 2 | 3 | 4 |
| I've been concentrating my efforts on doing something about the situation I'm in. | 1 | 2 | 3 | 4 |
| I've been saying to myself "this isn't real". | 1 | 2 | 3 | 4 |
| I've been using alcohol or other drugs to make myself feel better | 1 | 2 | 3 | 4 |
| I've been getting emotional support from others. | 1 | 2 | 3 | 4 |
| I've been giving up trying to deal with it. | 1 | 2 | 3 | 4 |
| I've been taking action to try to make the situation better. | 1 | 2 | 3 | 4 |
| I've been refusing to believe that it has happened. | 1 | 2 | 3 | 4 |
| I've been saying things to let my unpleasant feelings escape. | 1 | 2 | 3 | 4 |
| I’ve been getting help and advice from other people. | 1 | 2 | 3 | 4 |
| I've been using alcohol or other drugs to help me get through it. | 1 | 2 | 3 | 4 |
| I've been trying to see it in a different light, to make it seem more positive. | 1 | 2 | 3 | 4 |
| I’ve been criticizing myself. | 1 | 2 | 3 | 4 |
| I've been trying to come up with a strategy about what to do. | 1 | 2 | 3 | 4 |
| I've been getting comfort and understanding from someone. | 1 | 2 | 3 | 4 |
| I've been giving up the attempt to cope. | 1 | 2 | 3 | 4 |
| I've been looking for something good in what is happening. | 1 | 2 | 3 | 4 |
| I've been making jokes about it. | 1 | 2 | 3 | 4 |
| I've been doing something to think about it less, such as going to movies, watching TV, reading, daydreaming, sleeping, or shopping. | 1 | 2 | 3 | 4 |
| I've been accepting the reality of the fact that it has happened. | 1 | 2 | 3 | 4 |
| I've been expressing my negative feelings. | 1 | 2 | 3 | 4 |
| I've been trying to find comfort in my religion or spiritual beliefs. | 1 | 2 | 3 | 4 |
| I’ve been trying to get advice or help from other people about what to do. | 1 | 2 | 3 | 4 |
| I've been learning to live with it. | 1 | 2 | 3 | 4 |
| I've been thinking hard about what steps to take. | 1 | 2 | 3 | 4 |
| I’ve been blaming myself for things that happened | 1 | 2 | 3 | 4 |
| I've been praying or meditating | 1 | 2 | 3 | 4 |
| I've been making fun of the situation. | 1 | 2 | 3 | 4 |
