# Supplementary material for: Moderating Effect of Coping Strategies on the Association Between the Infodemic-Driven Overuse of Health Care Services and Cyberchondria and Anxiety: Partial Least Squares Structural Equation Modeling Study
Source: J Med Internet Res. 2024 Apr 9;26:e53417. doi: 10.2196/53417 (PMC11040441; doi:10.2196/53417)
Supplement: Multimedia Appendix 2 [file jmir_v26i1e53417_app2.docx]

Table Correlations between instruments (Pearson correlation coefficient)

|  | Overuse of HC | CSS-12 | GAD-7 | Coping. Problem | Coping. Emotion | Coping. Avoidant |
| --- | --- | --- | --- | --- | --- | --- |
| Infodemic | 0.21^***^ | 0.18^***^ | 0.19^***^ | -0.01 | -0.12^***^ | -0.14^***^ |
| Overuse of HC | - | 0.27^***^ | 0.11^***^ | 0.01 | -0.1^***^ | -0.12^***^ |
| CSS-12 | - | - | 0.28^***^ | -0.08^***^ | -0.28^***^ | -0.24^***^ |
| GAD-7 | - | - | - | -0.28^***^ | -0.22^***^ | -0.44^***^ |
| Coping.Problem | - | - | - | - | 0.23^***^ | 0.36^***^ |
| Coping.Emotion | - | - | - | - | - | 0.4^***^ |
| Coping.Avoidant | - | - | - | - | - | - |

Note: *** p<.001
